# Differentially Expressed miRNAs after GnRH Treatment and Their Potential Roles in FSH Regulation in Porcine Anterior Pituitary Cell

**DOI:** 10.1371/journal.pone.0057156

**Published:** 2013-02-22

**Authors:** Rui-Song Ye, Qian-Yun Xi, Qien Qi, Xiao Cheng, Ting Chen, Hongyi Li, Sanpha Kallon, Gang Shu, Song-Bo Wang, Qing-Yan Jiang, Yong-Liang Zhang

**Affiliations:** 1 Guandong Provincial Key Lab of Agro-Animal Genomics and Molecular Breeding, College of Animal Science, South China Agricultural University, Guangzhou, China; 2 School of Life Sciences, Longyan University, Longyan, China; Korea University, Korea, Republic of

## Abstract

Hypothalamic gonadotropin-releasing hormone (GnRH) is a major regulator of follicle-stimulating hormone (FSH) secretion in gonadotrope cell in the anterior pituitary gland. microRNAs (miRNAs) are small RNA molecules that control gene expression by imperfect binding to the 3′-untranslated region (3′-UTR) of mRNA at the post-transcriptional level. It has been proven that miRNAs play an important role in hormone response and/or regulation. However, little is known about miRNAs in the regulation of FSH secretion. In this study, primary anterior pituitary cells were treated with 100 nM GnRH. The supernatant of pituitary cell was collected for FSH determination by enzyme-linked immunosorbent assay (ELISA) at 3 hours and 6 hours post GnRH treatment respectively. Results revealed that GnRH significantly promoted FSH secretion at 3 h and 6 h post-treatment by 1.40-fold and 1.80-fold, respectively. FSHβ mRNA at 6 h post GnRH treatment significantly increased by 1.60-fold. At 6 hours, cells were collected for miRNA expression profile analysis using MiRCURY LNA Array and quantitative PCR (qPCR). Consequently, 21 up-regulated and 10 down-regulated miRNAs were identified, and qPCR verification of 10 randomly selected miRNAs showed a strong correlation with microarray results. Chromosome location analysis indicated that 8 miRNAs were mapped to chromosome 12 and 4 miRNAs to chromosome X. Target and pathway analysis showed that some miRNAs may be associated with GnRH regulation pathways. In addition, In-depth analysis indicated that 10 up-regulated and 3 down-regulated miRNAs probably target FSHβ mRNA 3′-UTR directly, including miR-361-3p, a highly conserved X-linked miRNA. Most importantly, functional experimental results showed that miR-361-3p was involved in FSH secretion regulation, and up-regulated miR-361-3p expression inhibited FSH secretion, while down-regulated miR-361-3p expression promoted FSH secretion in pig pituitary cell model. These differentially expressed miRNAs resolved in this study provide the first guide for post-transcriptional regulation of pituitary gonadotrope FSH secretion in pig, as well as in other mammals.

## Introduction

Hypothalamic-pituitary-gonadal axis(HPGA)governs almost all mammalian reproduction events, from fetal development, puberty to sexual maturity [1]. Gonadotrope cells of the anterior pituitary play a central role within this system, which respond to the hypothalamic gonadotropin-releasing hormone (GnRH) and secrete gonadotropins, luteinizing hormone (LH) and follicle-stimulating hormone (FSH) [2]. As a key hormone of HPGA system, FSH stimulates ovarian follicle growth and maturation in females, while regulating spermatogenesis of sertoli cells in male mammals [3]. GnRH regulation of FSH synthesis is modulated by multiple-layer factors. Numerous studies indicated that high pulsatility of GnRH induces the synthesis of LH while low pulsatility preferentially favors FSH production [4]–[6]. At transcription level, FSH secretion is mainly mediated by GnRH signaling pathway. GnRH binds to GnRHR located on gonadotrope and triggers a serial of sub-signalings including the second messenger signaling (cAMP, IP3 and calcium), PKC and MAPK (ERK1/2, JNK, p38) signaling pathways and subsequently induces expression of transcription factors, such as activator protein-1(AP-1, heterodimer consisting with c-Fos and c-Jun) and CREB, which are responsible for rate-limiting gene FSHβ transcription [7]–[10]. Recent studies demonstrate that the distinct pathway in GnRH pulse frequency is dependent on differential regulation of FSHβ transcription via CREB and ICER activation [11]. Pituitary transcription factor 1(Prop-1) and LIM homeodomain (LHX2) also stimulate porcine follicle stimulating hormone β subunit gene expression [12], [13].

miRNAs are a class of small non-coding RNAs (∼21 nt) that regulate mRNA translation at the post-transcriptional level, mainly by binding to the 3′-untranslated region (3′-UTR) of their targets [14]. HPGA-related miRNAs are widely reported, and several studies have shown that the miR-7 family is highly expressed in the hypothalamus and pituitary [15], [16]. In the pituitary, Dicer (−/−) mutant mice showed abnormal development and growth, which revealed the miRNAs function in animal pituitary. A variety of studies revealed miRNAs were expressed in tissues of HPGA system, and miRNAs may play a potential regulatory role in porcine ovary, testis and spermatogenesis [17]–[19]. In our previous study [20], the miRNA expression profiles of porcine pituitaries were also analyzed comprehensively by multiple methods. An increasing number of studies addressed the relationship between miRNAs and hormone induction and/or secretion [21]–[23]. However, as an important organ in controlling reproduction, details of how miRNAs regulate porcine FSH are still unknown. In the present study, miRNA expression profiles of primary porcine anterior pituitary cell during FSH secretion in response to GnRH were investigated by using miRNA microarray and qPCR. Bioinformatics analysis and functional experiments of differentially expressed miRNA were then conducted in order to reveal the potential role of miRNAs as new regulators in FSH secretion-related networks or pathways, especially the GnRH signaling pathway at the post-transcription level and to find out the precise mechanism of miRNAs in the anterior pituitary in reproduction control.

## Materials and Methods

### Ethics Statement

The animal slaughter experiments were conducted in accordance with the guidelines of Guangdong Province on the Review of Welfare and Ethics of Laboratory Animals approved by the Guangdong Province Administration Office of Laboratory Animals (GPAOLA).All animal procedures were conducted under the protocol (SCAU-AEC-2010-0416) approved by Institutional Animal Care and Use Committee (IACUC) of South China Agricultural University.

### Animal and primary cell culture

Six healthy male 7-day old piglets (Landrace) were slaughtered in legal program. Primary cell culture was performed as described in the previous studies [24], [25]. Briefly, under sterile conditions, pituitary glands were removed and the anterior lobe was immediately dissected from each pituitary gland. Six-anterior pituitary glands were washed with the Dulbecco's Modified Eagle's Medium/Nutrient Mixture F12 (DMEM/F12)(Gibco,US) supplemented with 100 IU/mL senicillin,100 µg/mL streptomycin, 2 mg/mL of BSA. Then, a pool of six-anterior pituitaries was minced for suspension in the same medium followed by centrifugation for 10 min at 2000 rpm. After the removal of the upper supernatant, the lower sliced fragment was incubated at 37°C in the DMEM/F12 containing 0.25% trypsin-EDTA (Gibco, US) and 0.25% collagenase type II in a flask with constant stirring for 30 min, then, the enzyme-digested pituitary suspension was centrifuged at 2000 rpm for 5 min, and the supernatant was discarded and the cell pellet was re-suspended with the medium. The cell suspension was then filtered through 75 µm nylon screens (200 meshes) to remove undigested tissue and cell aggregates and centrifuged at 2000 rpm for 10 min. The supernatant was discarded and the cell pellets were washed twice by the medium DMEM/F12 supplemented with antibiotics, then the cell pellets were diluted to 3×10^5^ live cells/mL with DMEM/F12 medium with 10% fetal calf serum (GIBCO, US), 100 IU/mL penicillin, 100 µg/mL streptomycin. Finally, 2 mL of cells suspension was seeded in 6-well plates (day of seeding = day 0 of culture), all cells were cultured at 37°C in a humidified atmosphere containing 5% CO_2_. Culture medium was replaced with fresh medium at 48 h after seeding, and experiments were performed on day 4.

### GnRH treatment and FSH determination

Before GnRH treatment, the medium was replaced with serum-free DMEM/F12 and incubated for 3 h at 37°C in a humidified atmosphere containing 5% CO_2_. Then the medium was discarded and plates were rinsed twice with serum-free DMEM/F12, and pituitary cells were cultured in 2 mL of fresh serum-free DMEM/F12 without antibiotics, but containing 100 nM GnRH (Gonadoliberin I (24–33): QHWSYGLRPG, Anaspec, USA). The same procedure was carried out with the control group except that GnRH was not added. All pituitary cells were re-incubated, and 200 µL of supernatant of pituitary cells were collected at 3 h and 6 h post GnRH treatment, respectively. At 6 h, pituitary cells were harvested and total RNA was extracted. FSH levels were measured using enzyme-linked immunosorbent assay (ELISA) according to the manufacturer's instructions (RapidBio, CA, USA) and total protein was determined for calibration by the BCA protein assay (Bioteke, Beijing, China).

### RNA extraction, miRNA microarray and qPCR

Total RNA was extracted from harvested cells by TRIzol reagent (Invitrogen, Carlsbad, CA, USA) following the manufacturer's instructions. Regarding both treatment and control groups, total RNA from six wells of pituitary cells was pooled together for miRNAs expression profile analysis. The miRNA expression was profiled using Exiqon's miRCURY LNA Array miRNA profiling services (Exiqon, Denmark).In brief, small RNAs were enriched by miRNeasy mini kit (QIAGEN),RNA quality and quantity were measured by using nanodrop spectrophotometer (ND-1000, Nanodrop Technologies) and RNA Integrity was determined by gel electrophoresis. Qualified RNAs were labeled using the miRCURY™ Hy3™/Hy5™ Power labeling kit (the treatment group was labeled with Hy3, the control was labeled with Hy5), and hybridized on the miRCURY™ LNA Array (v.11.0). Following the washing steps the slides were scanned using the Axon GenePix 4000B microarray scanner. Scanned images were then imported into GenePix Pro 6.0 software (Axon) for grid alignment and data extraction. miRNAs with two channel intensities>0 and SNR>1(or one of channel SNR>2) were chosen for further normalization. Expression data were normalized using the LOWESS (Locally Weighted Scatter plot Smoothing) regression algorithm (MIDAS, TIGR Microarray Data Analysis System), which can produce within-slide normalization to minimize the intensity-dependent differences between the dyes. Differentially expressed miRNAs were identified through Fold Change of 1.3 fold filtering.

The same RNA samples (no mix) were used for differentially-expressed miRNAs quantitative real-time PCR verification. In short, miRNAs were added as universal adaptor containing poly (A) tail and then reverse transcribed to cDNA using One Step PrimeScript® miRNA cDNA Synthesis Kit (Takara, Dalian, China) according to its instruction as described by a previous study [26]. cDNA was diluted by 5-folds with ddH_2_O, a final 20 µL volume qRT-PCR reaction was performed on STRATAGENE Mx3005P sequence detection system. PCR Reaction mix consisted of 2 µL cDNA, 10 µL of 2× SYBR Green PCR Master Mix (Toyobo, Osaka, Japan) and 1 mM of each primer. The thermal profile of real-time was as follows: 1 min at 95°C, 40 cycles of 15 s at 94°C and 15 s at corresponding annealing temperature (Tm), and 72°C for 40 s, followed by a quick denaturation at 95°C for 5 min, Tm, plus a slow ramp from Tm to 95°C to generate a melt curve to control the specificity of the amplified product. NTC (no template control) was set as negative control to each miRNA, all reactions were performed in triplicate. For all the differentially expressed miRNAs, the U6 small nuclear RNA was used as an internal control. 2^−ΔΔCt^ method was employed to quantify and normalize the expression data. Forward primers were designed by Primer 5.0, the reverse primer for miRNAs was Uni-miR qPCR Primer offered by kit, In addition, FSHβ mRNA expression was examined by using the same template, volume and thermal reaction, by using β-actin gene as control. Information about primers was listed in **Table S1**.

### Bioinformatics analysis

#### Chromosomal localization

According to mature sequence of differentially expressed miRNAs in microarray, miRNAs precursor sequences were obtained from miRBase release 18.0 (www.mirbase.org), then these sequences were mapped to pig genome (sscorfa9, www.ensembl.org/Sus_scrofa/), and the chromosomal localization map was plotted according to their relative position on chromosome.

#### Target prediction and pathway analysis

We predicted target genes of miRNA in pigs at genome level. In brief, the 3′-UTR sequences of porcine transcripts in whole genome were obtained from ensemble gene 66 (sscorfa9, www.ensembl.org/Sus_scrofa/). Mature differentially expressed miRNAs sequences were downloaded from miRBase release 18.0 (www.mirbase.org), and RNAhybrid software (www.bibiserv.techfak.uni-bielefeld.de/rnahybrid) was used to analyze miRNAs targets by using its own algorithm. Our prediction restricted perfect match of seed region (2–7 base of miRNAs 5-end, the G∶U match was permitted), due to importance of seed sequence for miRNA-mRNA binding [27]. In addition, we restricted less than −20 kcal/mol of low free energy in the binding of miRNA-mRNA. Furthermore, Genetrail online service (http://genetrail.bioinf.uni-sb.de/) [28] was used for pathway analysis according to all differentially expressed miRNA potential targets, and minimum number of genes of potential pathway affected by differentially expressed miRNAs is 20.

### Transient miRNAs transfection

Primary anterior pituitary cells were cultured in 6-well plate as described above (see “Animal and primary cell culture”). When the cells were 80–90% confluent, transfection experiment were conducted. Up-regulation of ssc-mir-361-3p was achieved by transfecting cells with 100 nM of synthetic RNA duplex (mimics, GenePharma, China) and negative control by Lipofectamine RNAiMAX (Invitrogen, Carlsbad, CA, USA) according to the manufacturer's protocol. ssc-mir-361-3p mimics sequences were as follows, 5′-CCCCCAGGUGUGAUUCUGAUUUGC-3′(sense), 5′-AAAUCAGAAUCACACCUGGGGGUU-3′ (antisense) and negative control sequence are 5′-UUCUCCGAACGUGUCACGUTT-3′(sense) and 5′-ACGUGACACGUUCGGGAATT-3′(antisense). Similarly, inhibition of ssc-miR-361-3p was achieved by transfecting cells with 100 nM of 2′-O methylated single-stranded ssc-miR-361-3p antisense oligonucleotides (inhibitors, GenePharma, China), ssc-miR-361-3p inhibitors sequence is 5′-GCAAAUCAGAAUCACACUGGGGG-3′ and inhibitors negative control (I-NC) sequence is 5′-CAGUACUUUUGUGUAGUACAA-3′. At 24 h following transfection, the supernatant of cells were collected for FSH hormone determination and cells were collected for RNA extraction to detect the expression of ssc-miR-361-3p by using stem-loop RT-qPCR [20]. The reverse transcription primer and qPCR primers for ssc-miR-361-3p were listed in **Table S1**.

### Statistical analysis

Statistical analysis of FSH determination, qPCR validation and correlation analysis were performed by SPSS 17.0 software. p<0.05 was considered significant, for pathway analysis, FDR was p-value adjustment for multiple testing, adjusted p<0.05 was considered significant.

## Results

### FSH secretion and its gene expression after GnRH treatment in porcine anterior pituitary cells

**Figure 1:**
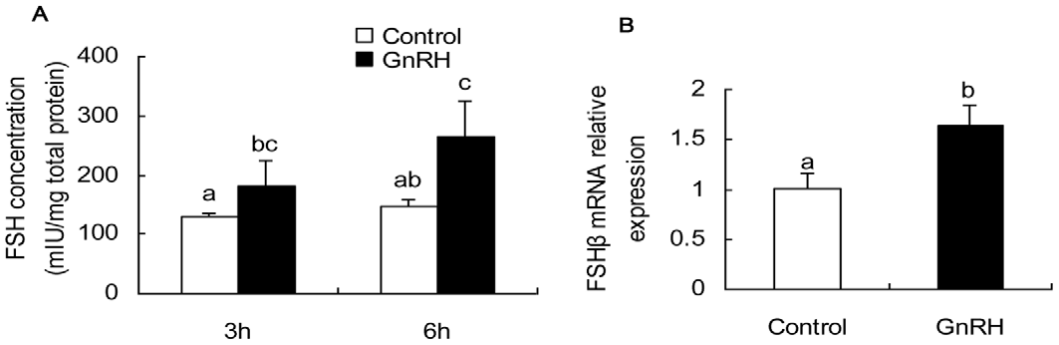
GnRH promoted FSH secretion at hormone and mRNA transcription. **A.** at 3 h post GnRH treatment, FSH concentration (mIU/mg total protein) in GnRH treatment group (180.00±41.08) was higher than that in the control group (128.11±7.67).At 6 h, FSH concentration in GnRH treatment was still higher relative to control group (264.74±60.92 vs. 147.7±21.44). Statistical significance was determined by ANOVA, followed by Tukey multiple comparisons test, p<0.05 was considered significant. **B.** GnRH increased FSHβ mRNA expression. Statistical significance was determined by Student's t test, p<0.05 was considered significant. Columns are means ± SD. The panels with different letters were considered statistically significant (p<0.05), N = 6.

In order to explore FSH secretion of porcine primary anterior pituitary cells in response to GnRH, cells were treated with 100 nM GnRH with low frequency (1 pulse in 6 hour), and FSH concentration in supernatant was measured by ELISA, followed by calibration using protein concentration. GnRH significantly promoted FSH secretion at 3 h and 6 h post-treatment. Compared with control group, FSH secretion in the GnRH group was significantly increased by 1.40-fold and 1.80-fold at 3 h and 6 h, respectively (**Figure 1A**). FSHβ mRNA at 6 h post GnRH treatment significantly increased (1.60-fold) relative to control (**Figure 1B**). Conclusively, GnRH promoted both FSH secretion and mRNA transcription in the porcine pituitary cell model.

### Differentially expressed miRNA profiles

A high–throughput miRNAs microarray was used to examine miRNA expression profiles of porcine pituitary cells after GnRH challenge. After normalization and quality assessment, a fold change of 1.3-fold (down-regulated miRNAs correspond to 0.77) was used to filter differentially expressed miRNAs. Twenty-one up-regulated (**Table 1**) and 10 down-regulated miRNAs (**Table 2**) were resolved from the microarray. According to seed sequences, miRNAs were classified into 28 miRNA families. Among all differentially expressed miRNAs, miR-133, miR-15/195 and let-7 families had two members. Interestingly, ssc-miR-361-5p and ssc-miR-361-3p shared the same precursor, so did ssc-miR-22-5p and ssc-miR-22-3p. More interestingly, ssc-miR-361-5p and ssc-miR-22-5p were up-regulated, while ssc-miR-361-3p and ssc-miR-22-3p were down-regulated.

**Table 1:** Up-regulated miRNA profiles of porcine pituitary cell after GnRH treatment.

miRNAs name	Fold-change	miRNAs family	Seed sequence
ssc-miR-133b	1.90	miR-133	UUGGUCC
ssc-miR-425-3p	1.90	miR-425/489	AUGACAC
ssc-miR-1307	1.85	miR-1307	CUCGGCG
ssc-miR-181d-5p	1.81	miR-181	ACAUUCA
ssc-miR-195	1.78	miR-15/16/195/424/497	AGCAGCA
ssc-miR-532-5p	1.77	miR-532/532-5p	AUGCCUU
ssc-let-7c	1.62	let-7/98	GAGGUAG
ssc-miR-130b	1.61	miR-130/301	AGUGCAA
ssc-miR-451	1.57	miR-451	AACCGUU
ssc-miR-105-2	1.54	miR-105.h	CAAAUGC
ssc-miR-19b	1.50	miR-19	GUGCAAA
ssc-miR-183	1.47	miR-183	AUGGCAC
ssc-miR-423-3p	1.46	miR-423/423-3p	GCUCGGU
ssc-miR-206	1.46	miR-1/206	GGAAUGU
ssc-miR-15a	1.42	miR-15/16/195/424/497	AGCAGCA
ssc-miR-22-5p	1.39	miR-22-5p/3568	GUUCUUC
ssc-miR-133a-3p	1.39	miR-133	UUGGUCC
ssc-miR-340	1.37	miR-340/340-5p	UAUAAAG
ssc-let-7a	1.37	let-7/98	GAGGUAG
ssc-miR-338	1.36	miR-338/338-3p	CCAGCAU
ssc-miR-361-5p	1.35	miR-361/361-5p	UAUCAGA

**Table 2:** Down-regulated miRNA profiles of porcine pituitary cell after GnRH treatment.

miRNAs name	Fold-change	miRNAs family	Seed sequence
ssc-miR-324	0.47	miR-324-5p	GCAUCCC
ssc-miR-708-5p	0.64	miR-28/28-5p/708	AGGAGCU
ssc-miR-151-3p	0.66	miR-151-3p	UAGACUG
ssc-miR-30e-3p	0.67	miR-30a-3p/30e	UUUCAGU
ssc-miR-152	0.69	miR-148/152	CAGUGCA
ssc-miR-21	0.70	miR-21/590-5p	AGCUUAU
ssc-miR-320	0.75	miR-320/320abcd	AAAGCUG
ssc-miR-22-3p	0.76	miR-22	AGCUGCC
ssc-miR-17-5p	0.76	miR-17-5p/20/93.mr/106/519.d	AAAGUGC
ssc-miR-361-3p	0.76	miR-361-3p	CCCCCAG

### Quantitative PCR

**Figure 2:**
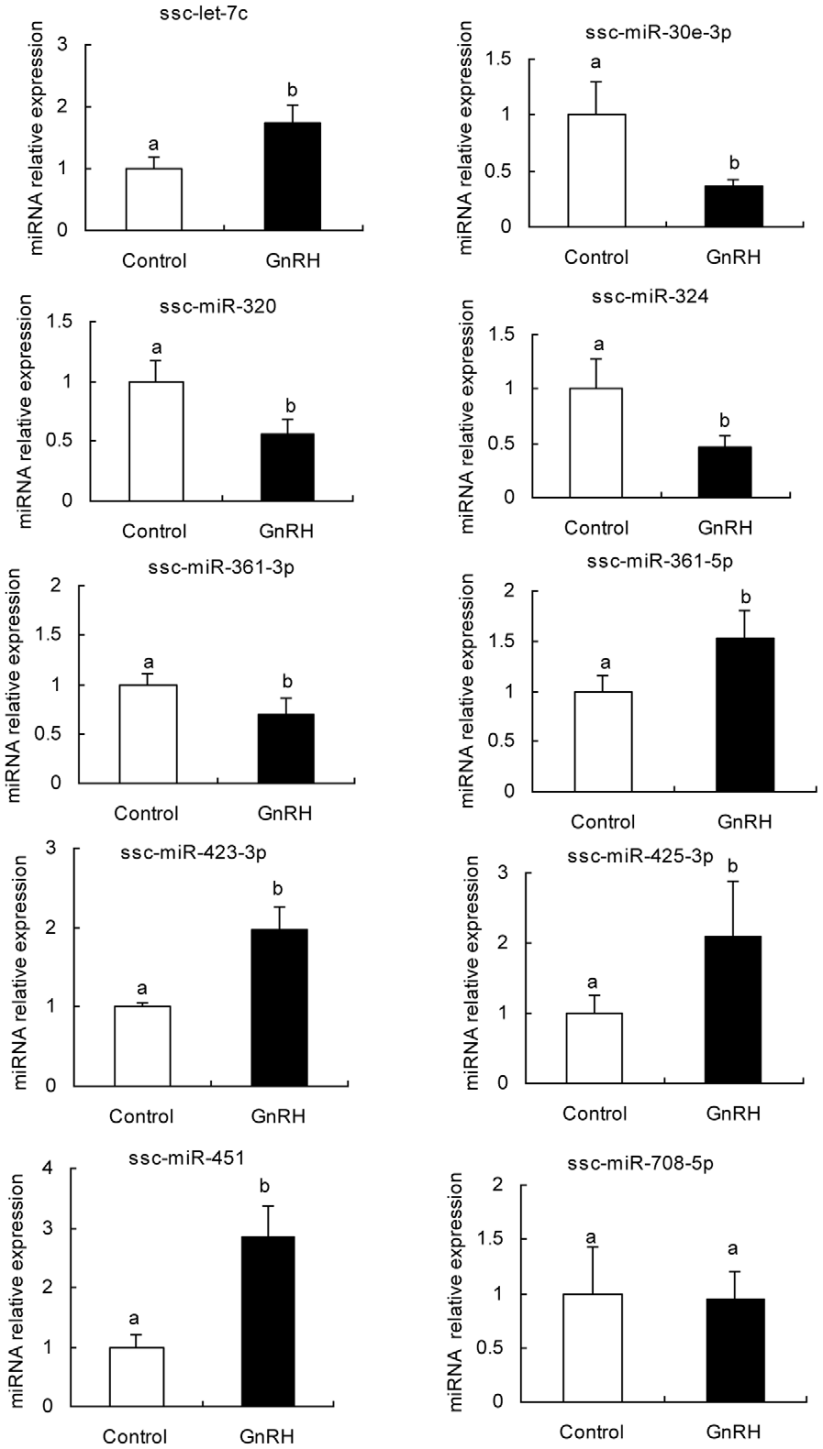
Relative expression of 10 differentially expressed miRNAs by revealed by quantitative PCR. ssc-let-7c, ssc-miR-361-5p, ssc-423-3p, ssc-miR-425-3p and ssc-miR-451 were up-regulated, while ssc-miR-30e-3p, ssc-miR-320, ssc-miR-324 and ssc-miR-361-3p were down-regulated after GnRH challenge. ssc-miR-708-5p was not significantly changed. Data in columns is means ± SD. statistical significance was determined by Student's t test, p<0.05 was considered significant. The panels with different letters were considered statistically significant (p<0.05), N = 6.

To validate the differentially expressed miRNAs revealed via microarray, 10 miRNAs were selected randomly (ssc-let-7c, ssc-miR-30e-3p, ssc-miR-320, ssc-miR-324, ssc-miR-361-3p, ssc-miR-361-5p, ssc-423-3p, ssc-miR-425-3p, ssc-miR-451 and ssc-miR-708-5p) for further analysis by using a special tail-added Real-time PCR method, taking U6 as an endogenous control. Interestingly, expression alterations of 10 miRNAs determined via qRT-PCR were completely consistent with microarray, except for ssc-miR-708-5p (no significant change). ssc-let-7c, ssc-miR-361-5p, ssc-423-3p, ssc-miR-425-3p and ssc-miR-451 were significantly up-regulated in the GnRH treatment group, while ssc-miR-30e-3p, ssc-miR-320, ssc-miR-324 and ssc-miR-361-3p were significantly down-regulated (**Figure 2**). In addition, these results showed that the expression levels determined by microarray analysis were consistent with those determined by quantitive RT-PCR. Pearson correlation analysis indicated a strong correlation between microarray and qRT-PCR (r = 0.89, p<0.01) in measurement of miRNAs expression.

### Chromosomal localization

**Figure 3:**
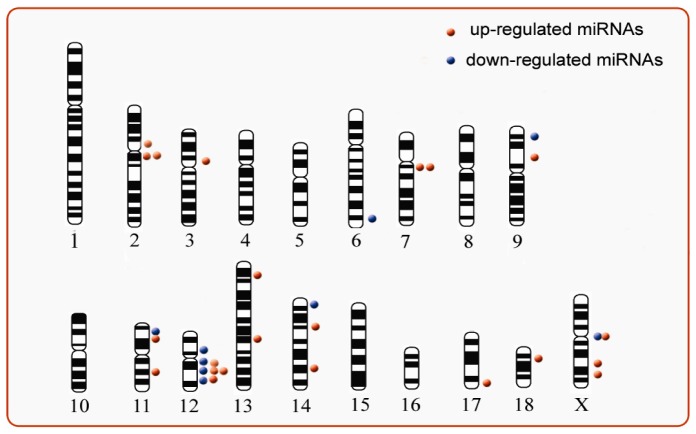
Chromosomal locus of differential expressed miRNAs. Red spots represent up-regulated miRNAs, while blue spots indicate down-regulated miRNAs. The spots in picture were plotted according to their relative position in chromosome length.

All differentially expressed miRNAs were mapped to pig chromosome (sscorfa9) according to the precursors of mature miRNAs. Consequently, 27 of 31 miRNAs were mapped to 12 different pig chromosomes (**Figure 3**, detail in **Table S2**) and 3 miRNAs (ssc-let-7a, ssc-miR-340 and ssc-miR-133a-3p) had two copies in the genome. Four miRNAs (ssc-miR-151-3p, ssc-miR-338, ssc-miR-532-5p and one of ssc-miR-133a-3p precursors) did not map to any chromosome in the database available. Two miRNAs (ssc-miR-133b, ssc-miR-206) formed a miRNA cluster and located on the same chromosome with an intergenic region of 3766bp, which suggests the miRNA cluster tends to be co-expressed. In addition, 4 miRNAs were mapped to chromosome X, including ssc-miR-19b, ssc-mir-105-2 as well as ssc-miR-361-5p and ssc-miR-361-3p, which were produced from the same precursor (ssc-miR-361). Most interestingly, at chromosome 12, over one quarter of all differentially expressed miRNAs (8 of 31) were mapped to region from 21743215 to 49872886 on chromosome 12. This suggests that GnRH stimulus may regulate transcription events at this region.

### Target prediction and pathway analysis

Target prediction is critical for miRNAs function exploration. Available online target-prediction tools, for example, Targetscan and PicTar, are limited in human and animal models such as mouse and rat. RNAhybrid is an effective tool for non-model animal miRNAs targets prediction [29]. By RNAhybrid algorithm, a total of 6174 potential targets (2887 genes with name annotation, **Table S3**) for 31 differentially expressed porcine miRNAs were predicted. Pathway analysis using Genetrail showed these predicted targets participate in 25 possible KEGG pathways (**Table 3**). These pathways are involved in immunity, cell communication, as well as pathways associated with GnRH regulation such as Jak-STAT signaling pathway [30], prostate cancer [31], and most importantly, the GnRH signaling pathway.

**Table 3:** Possible pathways affected by differentially expressed miRNAs.

KEGG Pathway Subcategory	Observed	P-value (FDR adjustment)
ssc4640:Hematopoietic cell lineage	26	7.33E-05
ssc1100:Metabolic pathways	199	7.33E-05
ssc190:Oxidative phosphorylation	25	7.33E-05
ssc4145:Phagosome	46	3.25E-04
ssc4060:Cytokine-cytokine receptor interaction	38	3.51E-04
ssc3040:Spliceosome	21	3.58E-03
ssc5010:Alzheimer's disease	37	8.05E-03
ssc5140:Leishmaniasis	27	8.05E-03
ssc4940:Type I diabetes mellitus	21	8.05E-03
ssc5416:Viral myocarditis	26	8.05E-03
ssc4142:Lysosome	22	8.99E-03
ssc4660:T cell receptor signaling pathway	28	1.56E-02
ssc5145:Toxoplasmosis	32	1.56E-02
ssc4062:Chemokine signaling pathway	38	1.83E-02
ssc5215:Prostate cancer	23	1.83E-02
ssc4630:Jak-STAT signaling pathway	26	2.63E-02
ssc5142:Chagas disease	36	2.69E-02
ssc5016:Huntington's disease	33	2.69E-02
ssc4514:Cell adhesion molecules (CAMs)	35	2.74E-02
ssc4144:Endocytosis	31	2.74E-02
ssc4810:Regulation of actin cytoskeleton	28	3.00E-02
ssc4912:GnRH signaling pathway	26	3.27E-02
ssc3320:PPAR signaling pathway	28	3.35E-02
ssc5414:Dilated cardiomyopathy	20	3.55E-02
ssc4612:Antigen processing and presentation	27	4.14E-02

**Figure 4:**
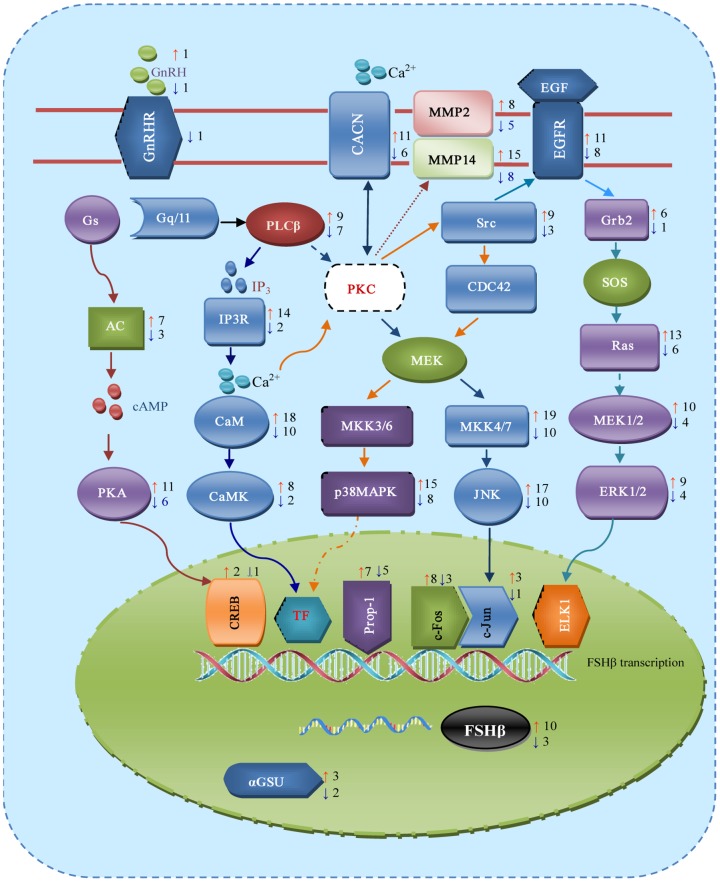
Predicted targets of miRNAs in GnRH signaling pathway. This figure was drawn according to KEGG pathway (ssc4912), red arrow near the box (general on the right) represents up-regulated miRNAs while blue arrow represents down-regulated miRNAs. The number near the arrow indicates number of miRNAs targeting this gene or its family. For example, 10 up-regulated miRNAs and 3 down-regulated miRNAs were predicted to target FSHβ mRNA 3′-UTR.TF indicates transcription factor involved in FSHβ transcription.

**Figure 5:**
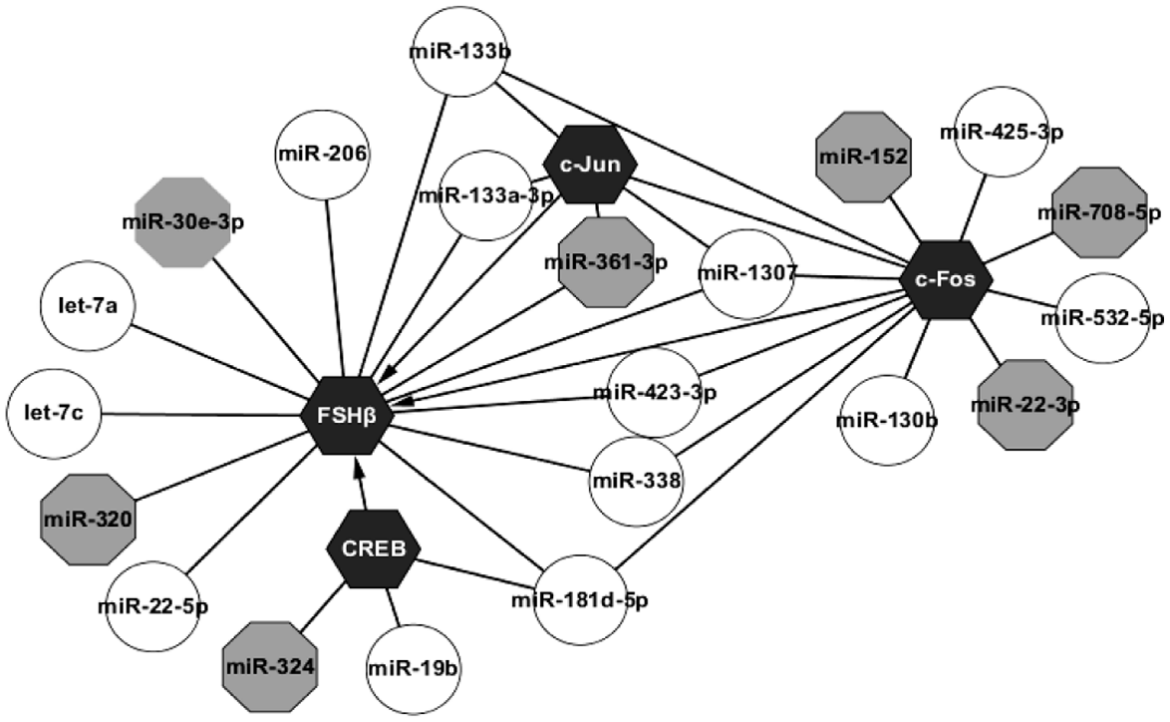
Predicted miRNAs against FSHβ, CREB, c-Fos and c-Jun. Round boxes (let-7a,let-7c,miR-22-5p,miR-19b,miR-181d-5p,miR-338,miR-423-3p,miR-133b, miR-206,miR-1307, miR-130b,miR-425-3p, and miR-532-5p) represent up-regulated miRNAs, gray octangle boxes (miR-30e-3p, miR-320, miR-324, miR-361-3p, miR-152, miR-708-5p, and miR-22-3p) represent down-regulated miRNAs, black hexagon boxes(FSHβ, CREB, c-Jun, c-Fos) indicate target genes. Arrows represent transcription factor-gene regulation, while dark lines represent miRNA regulation on mRNA except c-Jun and c-Fos.

Therefore, we further analyzed the interaction between miRNAs and mRNAs of the GnRH signaling pathway in detail (**Figure 4**). Interestingly, 1 miRNA (miR-361-3p) may target the GnRH signaling “switch” GNRHR. *in silico* analysis indicated that differentially expressed miRNAs likely target key molecules of GnRH signaling pathway branches (AC, PKA, PLCβ, IP3R, CaM, CaMK, JNK, Grb2, Ras, MEK1/2, ERK1/2 and p38MAPK) as well as important transcription factors(CREB, Prop1, c-Fos, c-Jun), and most importantly, the FSHβ mRNA 3′-UTR (All details were listed in **Table S4**). In addition, predicted miRNAs targeting CREB, AP-1(c-Jun and c-Fos) and FSHβ were illustrated in **Figure 5** **(detail in Table S4)**. Ten up-regulated and 3 down-regulated miRNAs were predicted to target FSHβ, 2 up-regulated and 1 down-regulated miRNAs may target CREB, 3 up-regulated and 1 down-regulated miRNAs target c-Jun, and 8 up-regulated and 3 down-regulated miRNAs likely target c-Fos. Our results revealed that one miRNA potentially targets several genes.

**Figure 6:**
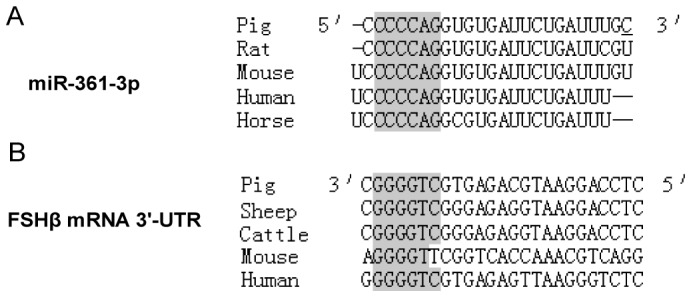
High conservation of miR-361-3p and its potential binding site in FSHβ mRNA 3′-UTR across mammalians. **A.** is alignment for mature miR-361-3p (named miR-361* in rat). **B.** is its binding site in FSHβ mRNA 3′-UTR across five species. Gray region represents seed sequence and its corresponding region.

Among the 13 possible miRNAs targeting FSHβ mRNA, interestingly, miR-361-3p had a highly conserved binding site in the FSHβ mRNA 3′-UTR (**Figure 6**) in mammals with perfect match in seed sequence across pig, sheep, cattle, mouse and human. Notably, miRBase data showed all miR-361-3p precursors of different organisms located on X chromosome, which suggested that this miRNA may play an important role in reproductive activity based on gender.

### ssc-miR-361-3p involved in FSH secretion regulation of porcine anterior pituitary cell

**Figure 7:**
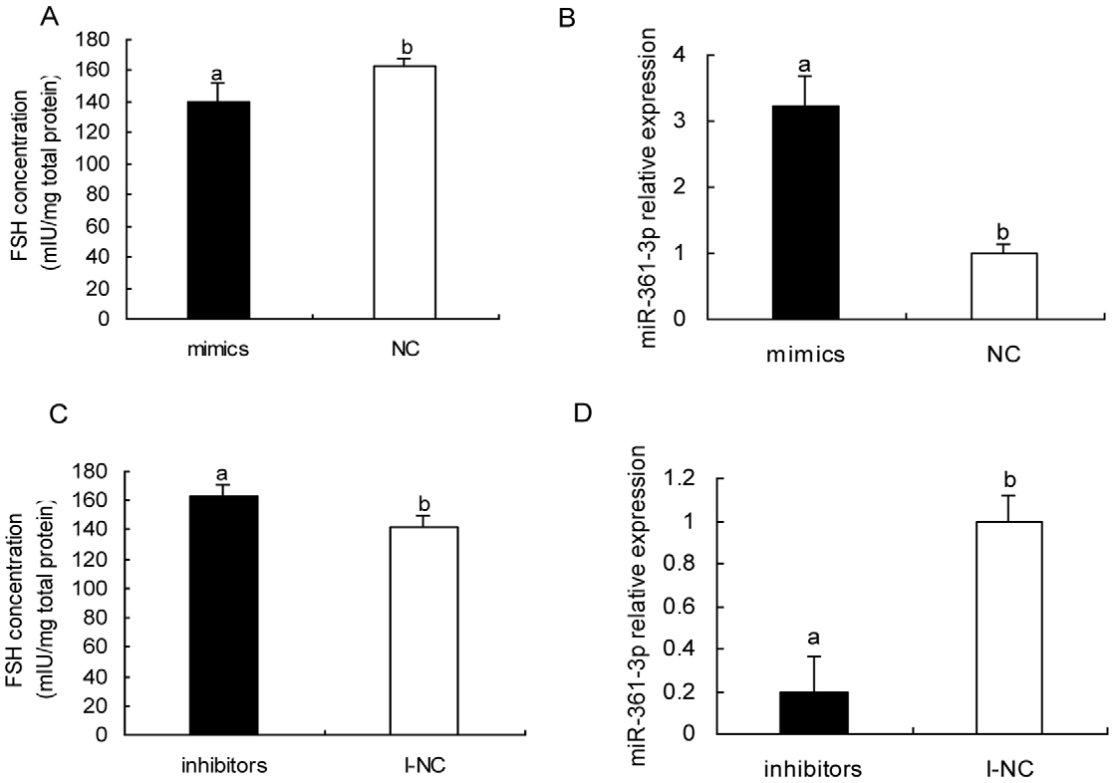
ssc-miR-361-3p involved in FSH secretion regulation. **A.** ssc-miR-361-3p mimics (mimics) and duplex negative control (NC) were separately transfected to primary pig pituitary cell, cell supernatant were collected for FSH determination at 24 h after transfection, ssc-miR-361-3p mimics led to significant decrease of FSH secretion by 14.10% (140.00±11.45 vs. 162.94±5.01 p<0.05). **B.** Transfection of miR-361-3p mimics increased miR-361-3p level in pituitary cell by 3.22-folds (p<0.05). **C.** miR-361-3p inhibitors increased FSH secretion by 15.07% compared with inhibitor negative control (I-NC) (163.31±7.04 vs. 141.91±7.81). **D.** Transfection of miR-361-3p inhibitors exhibited 80.87% decline of miR-361-3p level relative to I-NC (p<0.05). Data in columns is means ±SD, N = 6. Statistical significance between two groups was determined by Student's t test. The panels with different letters were considered statistically significant (p<0.05).

To further ascertain whether the alteration of ssc-miR-361-3p expression influences pituitary cell FSH secretion, ssc-miR-361-3p mimics and negative control were separately transfected to primary pig anterior pituitary cell. FSH concentration of cell supernatant were measured at 24 h after transfection, the result showed that up-regulation of ssc-miR-361-3p led to a significant decrease of FSH secretion by 14.1% (p<0.05) (**Figure 7A**), while ssc-miR-361-3p inhibitors significantly increased FSH secretion by 15.1% (p<0.05) (**Figure 7C**). In addition, the abundance of ssc-miR-361-3p was increased by mimics (**Figure 7B**) and decreased by inhibitors (**Figure 7D**). Interestingly, dramatic change of ssc-miR-361-3p expression caused only ∼15% FSH secretion increase or decrease, suggesting that ssc-miR-361-3p probably is a fine-tuned regulator in FSH secretion. These results indicated that ssc-miR-361-3p was involved in FSH secretion regulation in pig anterior pituitary cell model.

## Discussion

### Hormone secretion in response to GnRH

Primary pituitary cell culture has been frequently employed to study the effects of gonadotropin on FSH release [1], [6], [32], [33]. It has been proven that low frequency pulse (2–8 h) GnRH favors FSH secretion, while high frequency pulse GnRH induces LH secretion [4]. In this study, GnRH improved FSH secretion by an increase of 1.80-fold at 6 h after treatment. FSHβ mRNA expression was determined to increase by 1.60-fold. We also measured LH secretion, and no significant change was observed both at 3 h and 6 h (data not shown). Our results were quite similar with previous reports [4]–[6].

### Differentially expressed miRNAs after GnRH treatment

It has been well accepted that miRNAs are involved in hormone regulation. In primary rat pituitary cells, miR-325-3p was involved in stress-induced suppression of LH secretion [34].Yuen *et al* [23] analyzed microtranscriptome of murine immortalized gonadotrope cell lineage(LβT2 cell line) in response to 100 nM GnRH, and over 200 miRNAs were identified at 3 h after GnRH treatment, including miR-132/212 cluster. However, no data was shown on miRNAs involved in FSH or LH secretion. Moreover, the LβT2 cell line may have its own limitation since FSH secretion needs to be induced by activin A [35]. In this study, we tried to use pig anterior pituitary cell as a model to explore miRNAs involved in the regulation of anterior pituitary FSH secretion. Microarray revealed a total of 31 differentially expressed miRNAs, most of which had been detected in pig pituitary anterior lobe with high abundance in our previous study [20], such as let-7a, let-7c, miR-361-5p, miR-320, miR-21, miR-22, miR-152 and miR-15a. The significant alteration of those miRNAs strongly suggested that they likely play a critical role in pituitary-specific regulation of FSH secretion.

The differentially expressed miRNAs belonged to 28 families. Let-7, miR-15 and miR-133 families possessed at least two members, more interestingly, these miRNAs shared similar expression profile (up-regulated). Consistent with numerous studies [36], [37], miRNAs in the same family tend to share the same expression pattern. In the present study, miR-133b and miR-206 located closely on chromosome 7, and separated by only 3766 bp fragment. Both miRNAs were up-regulated after GnRH stimulation. Thus, it is reasonable to propose that they may be in the same cluster and co-expressed. Interestingly, chromosomal location analysis detected two couples of miRNAs, miR-22-5p/miR-22-3p and miR-361-5p/miR-361-3p, which come from the same precursor. These miRNAs had similar expression pattern, left arm miRNAs (-5p) were up-regulated while right arm (-3p) miRNAs were down-regulated. The results suggested the presence of post-transcription during the process from pri-miRNA to mature miRNAs in response to GnRH stimulation. It was reported that lin-28a played an important role in processing pri-let-7 to mature let-7 [38], [39]. We also revealed 8 differentially-expressed miRNAs located on chromosome 12 and 4 on chromosome X. Taken together, the results suggested the miRNAs located within close distance on a chromosome probably prefer to be co-expressed or co-regulated.

### miRNAs and FSH secretion

Target prediction of miRNAs by bioinformatics method is helpful to discover potential genes affected by miRNAs, and subsequently dissect miRNA regulatory networks or pathways. Up to date, RNAhybrid software, rather than Targetscan, PicTar and miRanda, is an effective miRNA target prediction tool for non-classical animal models [40].In this study, RNAhybrid was employed to predict potential targets of differentially expressed miRNAs, as a result, we obtained a total of 6174 possible transcripts (2887 with function annotation) as targets of 31 differentially expressed miRNAs. These results confirmed that one miRNA potentially targets hundreds of transcripts, and a single transcript may be targeted by multiple miRNAs. Subsequently, pathway analysis indicated that differentially expressed miRNAs might be involved in 25 pathways ranging from metabolism, immunity, disease, cell communication to hormone regulation. Most importantly, several pathways are associated with GnRH regulation, such as Jak-STAT signaling pathway [30], Prostate cancer [31], and the GnRH signaling pathway, which is the major pathway responsible for controlling FSH secretion.

Revealing target genes of differentially expressed miRNAs is the first step in explaining the increase of FSH at the post-transcription level. To better understand miRNAs' potential role in the regulation of FSH secretion, we pay more attention to predicting miRNA target genes involved in the regulation of the GnRH signaling pathway. By RNAhybrid prediction, 26 genes targeted by differentially expressed miRNAs cover all branches of the JNK, ERK1/2, p38 and second messenger pathways (cAMP, IP3, calcium). In the GnRH signaling pathway, AP-1 and CREB are two major transcription factors responsible for FSHβ gene transcription [1]. Differentially expressed miRNAs and factors such as AP-1 and CREB, most possibly, presented a complicated regulatory network. miRNAs may target FSHβ, and simultaneously its upstream regulatory genes (c-Fos, c-Jun, and CREB). For example, miR-133b is able to target FSHβ, c-Jun and c-Fos, while miR-361-3p may target c-Jun and FSHβ. In this regard, our study provided a comprehensive prediction for the potential role of miRNAs in the regulation of FSH synthesis and secretion.

Interestingly, miR-133b, miR-133a-3p and miR-206 were predicted to target FSHβ directly. Notably, miR-133b and miR-206 in one cluster shared the same expression pattern and the same target, indicating a novel regulatory mechanism of miRNAs. More interestingly, miR-133b, miR-133a-3p and miR-206 were firstly identified as muscle-specific miRNAs with a role in promoting the proliferation of myoblasts and inhibiting their differentiation [41]. GnRH plays a key role in gonadotrope cell proliferation [42], [43], increase of FSH secretion is partly due to the increase in population of gonadotrope, which may in part explain up-regulated miR-133 expression of gonadotrope response to GnRH stimulus. The let-7 family (let-7a, let-7c) was up-regulated in this study, suggesting their transcription and maturation might also be induced directly by gonadotropin-releasing hormone. Previous studies showed that 100 nM GnRH caused an increase of let-7a [23]. Prediction via software indicated that the let-7 family might target FSHβ with perfect match in seed sequence and high conservation among species (mouse and pig, data not shown), which indicated a potential role of the let-7 family in the regulation of FSH secretion. We also assessed conservation of miRNAs and their binding sites in FSHβ mRNA 3′-UTR across species (human, mouse, rat, pig and sheep). Notably, miR-361-3p and its target site in FSHβ mRNA were highly conserved across mammalian species. Most importantly, the miR-361-3p precursor was located on the X chromosome in all species, which suggests miR-361-3p is X-linked and plays a potential role in controlling reproductive event based on gender.

Some differentially expressed miRNAs are likely to take part in GnRH regulation of FSH secretion in the pituitary directly or indirectly. miR-15a and miR-195 are members of miR-15/16/195 family with up-regulated expression response to GnRH. Bottoni reported that miR-15a and miR-16-1 were down-regulated in pituitary adenomas [44]. Further studies demonstrated that miR-15a, miR-16-1, as well as miR-195 were important regulators of the cell cycle by targeting BCL2, DLEU2 [45], [46]. In the future, more experimental research is needed to explain how these miRNAs regulate FSH secretion.

### miR-361-3p and its potential role in FSH secretion regulation

Both miRNA array and qPCR results showed that miR-361-3p expression decreased during FSH secretion after GnRH treatment. Bioinformatic prediction showed that miR-361-3p potentially targets the FSHβ mRNA 3′-UTR. In the following trials of pig pituitary primary cells, increase of miR-361-3p expression inhibited FSH secretion. The results strongly suggested that miR-361-3p participated in FSH secretion regulation, most possibly by targeting the 3′-UTR of FSH mRNA. Our prediction also showed that miR-361-3p may have other targets involved in FSH secretion regulation. Up to date, little is known about miR-361-3p function. It was demonstrated that miR-361-3p was differentially expressed in several tumors or cancer [47]–[49]. Further experiments are needed to confirm the interaction between miR-361-3p and its potential targets. The detailed mechanisms responsible for FSH secretion remain to be elucidated in our next experiment.

In conclusion, our present study determined the underling differential expression of miRNA profiles involved in FSH secretion in response to GnRH. Bioinformatics analysis revealed that potential targets and pathways of miRNAs were altered significantly, which updated the GnRH signaling pathways regulation for FSH secretion. Most importantly, we identified a highly conserved X-linked miRNA, miR-361-3p, potentially targeting FSHβ. Furthermore, we showed for the first time that miR-361-3p negatively regulated FSH secretion in pig pituitary cell model. All these results are helpful in understanding the precise mechanism of FSH secretion in gonadotrope of the pituitary, even though more experimental research is needed to validate the authentic relationship between miRNAs and their targets.

## Supporting Information

Table S1 Primers for quantitative PCR.
(DOC)

Table S2 Chromosome location of miRNAs precursors.
(DOC)

Table S3 Potential target transcripts for differentially expressed miRNAs.
(XLS)

Table S4 Differentially expressed miRNAs in GnRH Signaling pathway.
(DOC)
