# Supplementary material for: Differentially Expressed miRNAs after GnRH Treatment and Their Potential Roles in FSH Regulation in Porcine Anterior Pituitary Cell
Source: PLoS One. 2013 Feb 22;8(2):e57156. doi: 10.1371/journal.pone.0057156 (PMC3579806; doi:10.1371/journal.pone.0057156)
Supplement: Table S1 — Primers for quantitative PCR. (DOC) [file pone.0057156.s001.doc]

| Name | Sequence(5→3) | Product Length(bp) | Tm |
| --- | --- | --- | --- |
| FSHβ | F: CCATCTCCCAATCTGTCTC  R: GCATTTAGTCCTTTCACCC | 177 | 58 |
| β-actin | F:CCAGCACCATGAAGATCAAGATC  R: ACATCTGCTGGAAGGTGGACA | 110 | 60 |
| U6 | F: CTCACTTCGGCAGCACATA  R:AACTCTTCACGATTTTGTCTGTC | 94 | 58 |
| ssc-let-7c | TGAGGTAGTAGGTTGTATGGTT | 79 | 60 |
| ssc-miR-320 | AAAAGCTGGGTTGAGAGGGCGA | 80 | 64 |
| ssc-miR-324 | CGCATCCCCTAGGGCATTG | 80 | 62 |
| ssc-miR-30e-3p | CTTTCAGTCGGATGTTTACAGC | 79 | 60 |
| ssc-miR-361-3p | CCCCCAGGTGTGATTCTGATTT | 81 | 60 |
| ssc-miR-361-5p | TTATCAGAATCTCCAGGGGTAC | 79 | 62 |
| ssc-miR-423-3p | AGCTCGGTCTGAGGCCC | 80 | 58 |
| Ssc-miR-425-3p | ATCGGGAATGTCGTGTCC | 79 | 60 |
| ssc-miR-451 | AAACCGTTACCATTACTGAGTT | 79 | 58 |
| ssc-miR-708-5p | AAGGAGCTTACAATCTAGCTGGG | 80 | 62 |
| ssc-miR-361-3p reverse transcription primer | GTCGTATCCAGTGCGTGTCGTGGAGTCGGCAATTGCACTGGATACGACGCAAATC | ---- | ---- |
| Stem-loop qRT-PCR Primer | F:ATCCAGTGCGTGTCGTGGA  R:CCCCCAGGTGTGATTC | 67 | 61 |

**Table S1. Primers for Real-time PCR**
