# Supplementary material for: Differentially Expressed miRNAs after GnRH Treatment and Their Potential Roles in FSH Regulation in Porcine Anterior Pituitary Cell
Source: PLoS One. 2013 Feb 22;8(2):e57156. doi: 10.1371/journal.pone.0057156 (PMC3579806; doi:10.1371/journal.pone.0057156)
Supplement: Table S2 — Chromosome location of miRNAs precursors. (DOC) [file pone.0057156.s002.doc]

**Table S2. Chromosome location of miRNAs precursor**

| miRNA precursor | Mature miRNAs | foldchange | Chromosome location |
| --- | --- | --- | --- |
| ssc-mir-181d | ssc-mir-181d | 1.8067439 | 2: 56889500-56889579 [+] |
| ssc-mir-340 | ssc-miR-340 | 1.3692322 | 2: 67820612-67820691 [+] |
| ssc-mir-340 | ssc-miR-340 | 1.3692322 | 2: 67919929-67920008 [+] |
| ssc-let-7a-2 | ssc-let-7a | 1.3691407 | 3: 38549760-38549851 [+] |
| ssc-mir-30e | ssc-miR-30e-3p | 0.6731788 | 6: 121824301-121824380[-] |
| ssc-mir-206 | ssc-miR-206 | 1.4620565 | 7: 53111391-53111470 [+] |
| ssc-mir-133b | ssc-mir-133b | 1.8978902 | 7: 53115236-53115315 [+] |
| ssc-mir-708 | ssc-miR-708-5p | 0.6422903 | 9: 13711347-13711426 [-] |
| ssc-let-7a-1 | ssc-let-7a | 1.3691407 | 9: 47697398-47697477 [-] |
| ssc-mir-15a | ssc-mir-15a | 1.4221705 | 11: 17602978-17603056 [+] |
| ssc-mir-17 | ssc-miR-17-5p | 0.7589734 | 11: 60972458-60972534 [+] |
| ssc-mir-19b-1 | ssc-miR-19b | 1.4985592 | 11: 60973040-60973119 [+] |
| ssc-mir-152 | ssc-miR-152 | 0.6861971 | 12: 21743215-21743294 [+] |
| ssc-miR-21 | ssc-miR-21 | 0.7033854 | 12: 34201565-34201656 [+] |
| ssc-miR-451 | ssc-miR-451 | 1.5715925 | 12: 42820766-42820830 [-] |
| ssc-miR-423 | ssc-miR-423-3p | 1.4623717 | 12: 44150500-44150579 [+] |
| ssc-mir-22 | ssc-miR-22-5p | 1.3933029 | 12: 45319043-45319122 [+] |
| ssc-mir-22 | ssc-miR-22-3p | 0.7550159 | 12: 45319043-45319122 [+] |
| ssc-mir-195 | ssc-mir-195 | 1.7784682 | 12: 49575884-49575963 [+] |
| ssc-miR-324 | ssc-miR-324 | 0.4693294 | 12: 49872807-49872886 [+] |
| ssc-let-7c | ssc-let-7c | 1.6172011 | 13: 130665021-130665114 [+] |
| ssc-mir-425 | ssc-mir-425-3p | 1.8663805 | 13: 26605151-26605230 [-] |
| ssc-miR-1307 | ssc-miR-1307 | 1.8454029 | 14: 119440203-119440282 [-] |
| ssc-miR-130b | ssc-miR-130b | 1.6121387 | 14: 51318779-51318858 [-] |
| ssc-miR-320 | ssc-miR-320 | 0.7546887 | 14: 6473891-6473970 [-] |
| ssc-mir-133a-1 | ssc-miR-133a-3p | 1.3869355 | 17: 64053943-64054045 [-] |
| ssc-mir-183 | ssc-mir-183 | 1.4686586 | 18: 17172238-17172307 [-] |
| ssc-mir-19b-2 | ssc-miR-19b | 1.4985592 | X: 108212546-108212625 [-] |
| ssc-mir-105-2 | ssc-mir-105-2 | 1.5363514 | X: 121720259-121720338 [+] |
| ssc-miR-361 | ssc-miR-361-5p | 1.3468151 | X: 67904186-67904265 [-] |
| ssc-miR-361 | ssc-miR-361-3p | 0.7643755 | X: 67904186-67904265 [-] |
| ssc-mir-151 | ssc-miR-151-3p | 0.6583531 | unknown |
| ssc-mir-338 | ssc-miR-338 | 1.3635822 | unknown |
| ssc-mir-133a-2 | ssc-miR-133a-3p | 1.3869355 | unknown |
| sc-miR-532 | ssc-miR-532-5p | 1.7745822 | unknown |
