# Supplementary material for: Differentially Expressed miRNAs after GnRH Treatment and Their Potential Roles in FSH Regulation in Porcine Anterior Pituitary Cell
Source: PLoS One. 2013 Feb 22;8(2):e57156. doi: 10.1371/journal.pone.0057156 (PMC3579806; doi:10.1371/journal.pone.0057156)
Supplement: Table S4 — Differentially expressed miRNAs in GnRH Signaling pathway. (DOC) [file pone.0057156.s004.doc]

**Table S4. Differentially** expressed miRNAs in GnRH Signaling pathway.

| miRNAs | Targets in GnRH signaling pathway |
| --- | --- |
| ssc-let-7a | CACNA1D,CALM1,EGFR,ERK2,fshb,grb2,ITPR3,LOC100517106,MAP3K1,MAP3K3,MAPK14,MAPK8,MAPK9,mmp2, nras, pla2g2d,plg2g2f,PTK2B,SRC |
| ssc-let-7c | CACNA1D,calm1,calm3,egfr,erk2,fshb,grb2,ITPR3,LOC100517106,LOC100518251, MAP3K1, MAP3K3, MAP3K4, mapk12, MAPK14, MAPK8, MAPK9, mmp2, nras, pla2g2d, plg2g2f, PRKACA, PTK2B,SRC |
| ssc-miR-105-2 | calm3,camk2g,egfr,MAP3K3,MAPK8 |
| ssc-miR-1307 | ADCY8,calm1,calm3,cjun,egfr,erk2,grb2,ITPR3,LOC100514711,LOC100517106,LOC100520418,map2k1,MAP3K3,MAP3K4,mapk12,MAPK8,MAPK9,mmp14,mmp2,fshb,pla2g2d,PLCB2,PLCB3,plg2g2f,PRKACA,PTK2B, SRC,prop-1 |
| ssc-miR-130b | CACNA1D,calm1,calm3,erk2,map2k1,MAP3K1,MAP3K3,MAP3K4,MAPK14,MAPK9,mmp14,mmp2,nras, pla2g12b, pla2g2d,plg2g2f |
| ssc-miR-133a-3p | ADCY8,CACNA1D,calm1,calm3,camk2d,cjun,egfr ITPR3,LOC100514711,LOC100520418, ap2k1, MAP3K1, MAP3K3,mapk12,MAPK14,MAPK8,mmp14,fshb, PLCB3,plg2g2f, PRKACA, PTK2B,SRC |
| ssc-miR-133b | CACNA1D,calm1,calm3,camk2d,cjun,egfr,ITPR3,LOC100514711,,LOC100517106,LOC100518251,LOC100520418,MAP3K1,MAP3K3,mapk12,MAPK14,MAPK8,MAPK9,mmp14,mmp2,fshb,PLCB3,plg2g2f,PRKACA,PTK2B,SRC,pROP-1 |
| ssc-miR-151-3p | ADCY8,CACNA1D,calm1,calm3,grb2,map2k1,MAP3K3,mapk12,MAPK14,MAPK8,MAPK9,plg2g2f,PTK2B,SRC, Prop-1 |
| ssc-miR-152 | calm1,calm3,MAP3K3,MAP3K4,MAPK14,MAPK9,mmp14,mmp2,nras,PLCB3,plg2g2f,  PRKACA |
| ssc-miR-15a | ADCY8,calm1,ITPR3,LOC100514711,map2k1,MAP3K3,MAPK14,MAPK8,MAPK9,mmp14,  PRKACA, PTK2B |
| ssc-miR-17-5p | CACNA1D,calm3,egfr,erk2, MAP3K1,MAP3K3,MAP3K4,MAPK8,MAPK9, mmp14,mmp2 |
| ssc-miR-181d-5p | atf4, calm1, calm3, egfr, ITPR3, MAP3K1, MAP3K3, MAPK14,MAPK8,MAPK9,mmp14, fshb, PLCB2, plg2g2f |
| ssc-miR-183 | CACNA1D,calm1,ITPR3,LOC100518251,map2k1,MAP3K3,MAP3K4,mapk12,MAPK14,mmp14,pla2g4b,PLCB2,plg2g2f,PTK2B,SRC,calm1,ITPR3,map2k1,MAP3K3, MAPK8,MAPK9, mmp14, PRKACA,PTK2B, pROP-1 |
| ssc-miR-195 | calm1,ITPR3,map2k1,MAP3K3,MAPK8,MAPK9,mmp14, PRKACA,PTK2B |
| ssc-miR-19b | atf4,MAP3K1,MAP3K3 |
| ssc-miR-206 | ADCY8,CACNA1D,calm1,camk2d,egfr,LOC100514711,LOC100518251,map2k1,MAP3K1,MAP3K3,MAPK8,MAPK9,mmp14,nras,fshb,pla2g2d |
| ssc-miR-21 | ADCY8,calm1,egfr,LOC100516581,MAP3K1,MAPK9,mmp14 |
| ssc-miR-22-3p | calm1,calm3,camk2g,egfr,erk2,ITPR3,MAP3K1,MAP3K3,MAPK10,MAPK14,MAPK8,mmp14,mmp2,PLCB2,PTK2B, Prop-1 |
| ssc-miR-22-5p | ADCY8,CACNA1D,calm1,calm3,camk2g,egfr,erk2,grb2,ITPR3,LOC100514711,LOC100517106,LOC100518251,LOC100520418,MAP3K1,MAP3K3,mapk12,MAPK14,MAPK9,mmp14,mmp2,fshb,pla2g2d,PLCB3,plg2g2f,,PRKACA,PTK2B, SRC |
| ssc-miR-30e-3p | CACNA1D,calm1,calm3,egfr,gnrh1,LOC100518251,LOC100520418,map2k1,MAP3K3,MAP3K4,mapk12,MAPK9, mmp14,fshb,PLCB3,plg2g2f,PRKACA |
| ssc-miR-320 | CACNA1D,calm1,calm3,egfr,erk2,LOC100517106,LOC100518251,MAP3K1,MAP3K3,mapk12,MAPK9,fshb,plg2g2f, PRKACA,PTK2B, Prop-1 |
| ssc-miR-324 | atf4,calm1,calm3,camk2d,egfr,ITPR3,LOC100517106,LOC100520418,MAP3K3,mapk12,MAPK14,MAPK9,mmp14,mmp2,pla2g4b,PLCB2,plg2g2f,PRKACA |
| ssc-miR-338 | calm1,calm3,MAP3K3,MAPK8,MAPK9,mmp14,fshb,plg2g2f,PRKACA |
| ssc-miR-340 | ITPR3,MAPK14 |
| ssc-miR-361-3p | ADCY8,CACNA1D,calm3,cjun,egfr,LOC100514711,LOC100518251,map2k1,MAP3K3,mapk12,MAPK8,mmp14,fshb,pla2g4b,PLCB2,PLCB3,plg2g2f,PRKACA,PTK2B,SRC,Gnrhr , Prop-1 |
| ssc-miR-361-5p | CACNA1D,calm1,calm3,erk2,grb2,LOC100518251,MAP3K3,MAP3K4,MAPK14,MAPK9,mmp14,mmp2,pla2g12b,plg2g2f,Prop-1 |
| ssc-miR-423-3p | ADCY8,CACNA1D,calm1,calm3,camk2d,camk2g,egfr,erk2,grb2,ITPR3LOC100514711,LOC100518251,LOC100520418,map2k1,MAP3K1,MAP3K3,MAP3K4,MAPK14,MAPK8,MAPK9,mmp14,mmp2,fshb,pla2g12b,pla2g2d,PLCB2,PLCB3,plg2g2f,PRKACA,PTK2B,SRC,Prop-1 |
| ssc-miR-425-3p | ADCY8,CACNA1D,calm1,calm3,camk2d,egfr,erk2,gnrh1,ITPR3,LOC100514711,LOC100517106,map2k1,MAP3K3,MAP3K4,mapk12,MAPK14MAPK9,mmp14,nras,pla2g12b,pla2g2d,pla2g4b,PLCB3,plg2g2f,PRKACA,SRC ,PROP-1 |
| ssc-miR-451 | prop-1 |
| ssc-miR-532-5p | calm1,camk2g,erk2,ITPR3,LOC100514711,LOC100517106,LOC100518251,map2k1,MAP3K3,mapk12,MAPK14,MAPK8,MAPK9,mmp14,PLCB2,plg2g2f,PRKACA,PTK2B |
| ssc-miR-708-5p | CACNA1D,calm1,calm3,egfr,erk2,LOC100514711,LOC100517106,LOC100518251,map2k1,MAP3K1,MAP3K3,MAP3K4,mapk12,MAPK14,MAPK8,MAPK9,mmp14,mmp2,nras,pla2g12b,pla2g2d,PLCB2,plg2g2f,PRKACA,SRC, Prop-1 |
